# 2023 updated MASCC/ESMO consensus recommendations: prevention of nausea and vomiting following high-emetic-risk antineoplastic agents

**DOI:** 10.1007/s00520-023-08221-4

**Published:** 2023-12-21

**Authors:** Jørn Herrstedt, L Celio, PJ Hesketh, L Zhang, R Navari, A Chan, M Saito, R Chow, M Aapro

**Affiliations:** 1 Department of Clinical Oncology, Zealand University Hospital, Sygehusvej 10, DK-4000 Roskilde, Denmark; 2 Institute of Clinical Medicine, University of Copenhagen, Copenhagen, Denmark; 3 Milan, Italy; 4 Division of Hematology Oncology, Lahey Hospital and Medical Center, Burlington, MA USA; 5 State Key Laboratory of Oncology in South China, Collaborative Innovation Center for Cancer Medicine, Sun Yat-sen University Cancer Center, Guangzhou, China; 6 World Health Organization, Birmingham, Alabama USA; 7 Department of Clinical Pharmacy Practice, School of Pharmacy & Pharmaceutical Sciences, University of California Irvine, Irvine, CA USA; 8 Department of Breast Oncology, Juntendo University School of Medicine, Tokyo, Japan; 9 Temerty Faculty of Medicine, University of Toronto, Toronto, ON Canada; 10 Genolier Cancer Center, Genolier, Switzerland

**Keywords:** High-emetic-risk chemotherapy, HEC, Antiemetics, Nausea, Vomiting, Guideline

## Abstract

**Purpose:**

This systematic review updates the MASCC/ESMO recommendations for high-emetic-risk chemotherapy (HEC) published in 2016–2017. HEC still includes cisplatin, carmustine, dacarbazine, mechlorethamine, streptozocin, and cyclophosphamide in doses of > 1500 mg/m^2^ and the combination of cyclophosphamide and an anthracycline (AC) in women with breast cancer.

**Methods:**

A systematic review report following the PRISMA guidelines of the literature from January 1, 2015, until February 1, 2023, was performed. PubMed (Ovid), Scopus (Google), and the Cochrane Database of Systematic Reviews were searched. The literature search was limited to randomized controlled trials, systematic reviews, and meta-analyses.

**Results:**

Forty-six new references were determined to be relevant. The main topics identified were (1) steroid-sparing regimens, (2) olanzapine-containing regimens, and (3) other issues such as comparisons of antiemetics of the same drug class, intravenous NK_1_ receptor antagonists, and potentially new antiemetics. Five updated recommendations are presented.

**Conclusion:**

There is no need to prescribe steroids (dexamethasone) beyond day 1 after AC HEC, whereas a 4-day regimen is recommended in non-AC HEC. Olanzapine is now recommended as a fixed part of a four-drug prophylactic antiemetic regimen in both non-AC and AC HEC. No major differences between 5-HT_3_ receptor antagonists or between NK_1_ receptor antagonists were identified. No new antiemetic agents qualified for inclusion in the updated recommendations.

## Introduction

The risk of nausea and vomiting following antineoplastic therapy depends on the emetic risk potential of the antineoplastic therapy, patient demographics such as sex (women are at a higher risk than men) and age (younger patients have a higher risk than older), and the antiemetic prophylaxis prescribed.

The emetic risk of antineoplastic agents administered intravenously (i.v.) is defined as the risk of vomiting within the first 24 h after the start of antineoplastic therapy in patients who did not receive antiemetic prophylaxis. High emetic risk is defined as a risk of more than 90% of vomiting. High-emetic-risk antineoplastic agents administered intravenously include cisplatin, carmustine, dacarbazine, mechlorethamine, streptozocin, and cyclophosphamide in doses of > 1500 mg/m^2^ and the combination of cyclophosphamide and an anthracycline (AC) in women with breast cancer. All these are chemotherapeutic agents and are referred to as high-emetic-risk chemotherapy (HEC).

Very few data on the emetic risk potential of orally administered antineoplastic agents exist, and the emetic risk potential refers to the risk during the entire treatment period rather than the first 24 h.

This manuscript is a systematic review and update of the MASCC/ESMO recommendations for high-emetic-risk antineoplastic agents published in 2016–2017 [1, 2].

## Methods

**Fig. 1:**
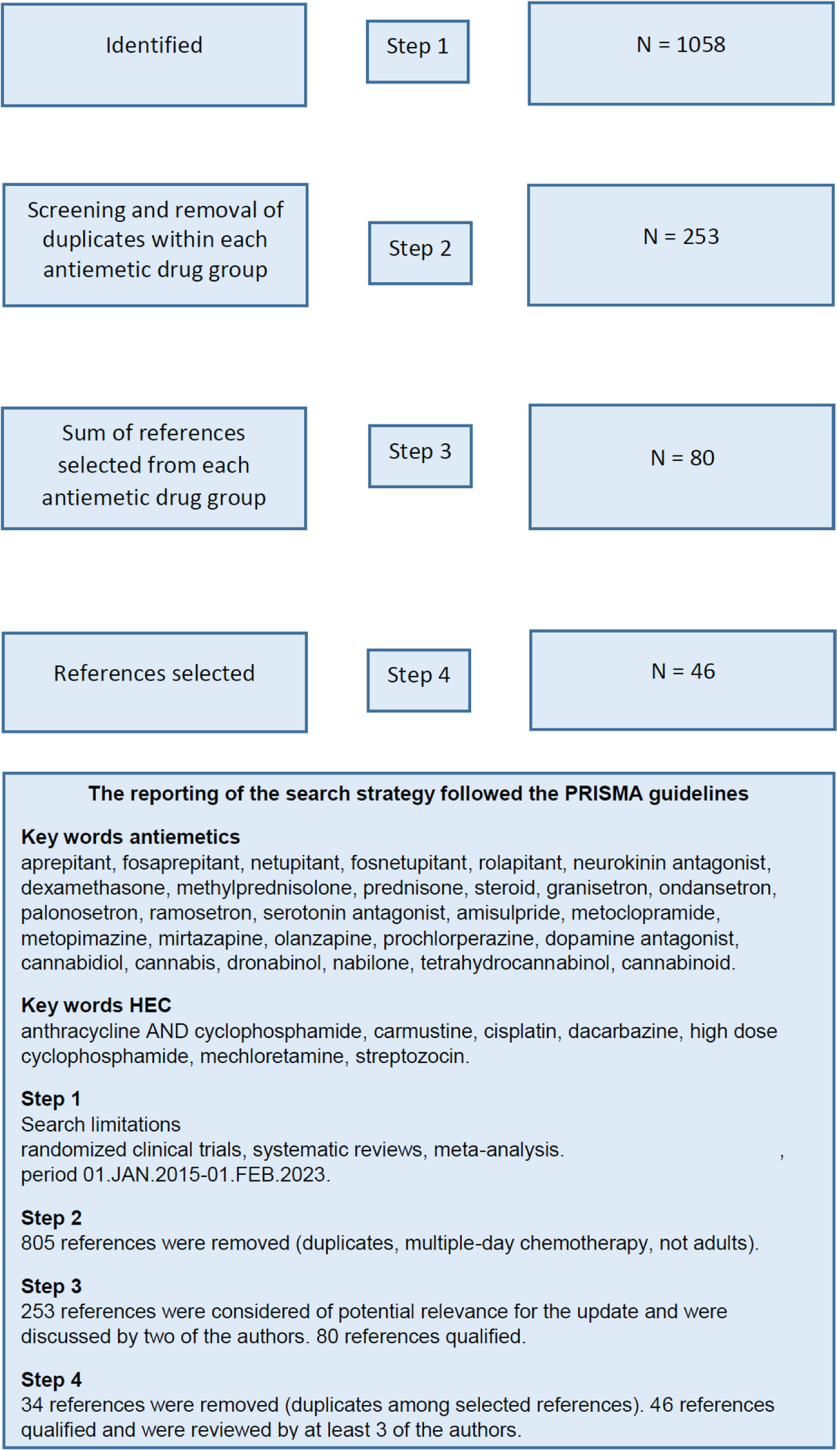
Flowchart literature search: HEC and antiemetics

A literature search was conducted from January 1, 2015, through February 1, 2023. PubMed (Ovid), Scopus (Google), and the Cochrane Database of Systematic Reviews were searched. The reporting of literature search followed the PRISMA guidelines [3]. The seven HEC agents were used as keywords and paired with each of the available antiemetics within the five antiemetic drug groups (neurokinin (NK)_1_ receptor antagonists, serotonin (5-HT)_3_ receptor antagonists, corticosteroids, dopamine (D)_2,3_ receptor antagonists, and cannabinoids). For example, the search terms for cisplatin were as follows: cisplatin AND aprepitant OR netupitant OR rolapitant OR fosaprepitant OR fosnetupitant OR neurokinin antagonist; cisplatin AND ondansetron OR granisetron OR palonosetron OR ramosetron OR serotonin antagonist; cisplatin AND dexamethasone or methylprednisolone or prednisolone or steroid; cisplatin AND metoclopramide OR domperidone OR metopimazine OR prochlorperazine OR olanzapine OR amisulpride OR dopamine antagonist; cisplatin AND cannabis OR tetrahydrocannabinol OR nabilone OR dronabinol OR cannabidiol OR cannabinoid. The search was limited to randomized controlled trials, systematic reviews, and meta-analyses. The number of results identified from literature search and determined to be relevant is summarized as a PRISMA flow diagram in Fig. 1. For the distribution of selected references in each of the antiemetic drug groups, see Table 1.

**Table 1:** Status literature search high-emetic-risk antineoplastic agents

Antiemetic drug group	References included in the 2023 update	Final number (duplicates deleted)
NK_1_ receptor antagonist	25	46
5-HT_3_ receptor antagonist	35	
Dopamin_2,3_ receptor antagonists and multi-receptor targeting agents (e.g., olanzapine)	14	
Corticosteroids	6	
Cannabinoids	0	
Total	80	

## Results

A total of 80 references were identified as relevant for further full-text review after reading abstracts of all 1058 references. The 80 references were distributed as follows: NK_1_ receptor antagonist (*n* = 25), 5-HT_3_ receptor antagonists (*n* = 35), D_2,3_ receptor antagonists (*n* = 14), corticosteroids (*n* = 6), and cannabinoids (*n* = 0). After removal of duplicates, 46 references qualified for consideration by the guideline committee, in the context of updating this guideline [2, 4–48]. The references were divided into three categories according to the quality of the study and the potential to change the guideline.

- *Category 1* [*4, 9, 12, 13, 20, 22, 24, 25, 30, 34, 38, 39, 41, 44, 48*]:
- References have the potential to change the guidelines and are described in detail in the manuscript.

- *Category 2* : [*5-8, 10, 11, 14, 15, 17-19, 21, 23, 26-28, 31-33, 35-37, 40, 42, 43, 45, 47*]:
- References are supportive for category 1 references and are described briefly in the manuscript.

- *Category 3* [*3, 16, 29, 46*]:
- References about new agents or minor studies in new settings may be hypothesis generating. These references are mentioned in the manuscript.

The main topics identified were (1) steroid-sparing regimens, (2) olanzapine-containing regimens, and (3) other issues such as comparisons of antiemetics of the same drug class, intravenous NK_1_ receptor antagonists, and potentially new antiemetics.

### Steroid-sparing regimens

The literature search identified six references qualifying for inclusion in the current update. These included two original studies [8, 22], a combined analysis of these two studies [7], a sub-analysis [6] of one of the studies [8], and two meta-analysis [5, 31] of which one [31] included a systematic review. A meta-analysis from 2019 including eight studies concluded that a single day of dexamethasone (DEX) is as good as a 3-day regimen in patients receiving moderately emetogenic chemotherapy (MEC) or AC chemotherapy [5]. Another systematic review and meta-analysis from 2019 including five studies and using a non-inferiority margin of −8% confirmed non-inferiority of a 1-day DEX regimen compared to a 3-day DEX regimen in MEC and AC patients [31]. This was further investigated in a randomized, double-blinded, placebo-controlled, non-inferiority trial including 396 patients [22]. Patients in this trial received cisplatin-based (> 50 mg/m^2^) or AC chemotherapy. Patients were randomized to receive either DEX day 1 (12 mg i.v.) plus placebo days 2–3 or DEX 12 mg i.v. day 1 followed by DEX 8 mg days 2–3. All patients also received palonosetron 0.75 mg i.v. plus aprepitant 125 mg p.o. day 1 followed by 80 mg p.o. days 2–3 or fosaprepitant 150 mg i.v. day 1. Patients were stratified for age and chemotherapy (cisplatin versus AC). The primary end point was complete response (CR) in the overall period (defined as no emetic episodes and no use of rescue medication days 1–5 after chemotherapy), and the non-inferiority margin was 15%. CR was 46.9% (3 days of DEX) versus 44% (1 day of DEX), *p* = 0.007 (95% CI, −12.6 to 6.8%). A subgroup analysis of patients receiving AC confirmed non-inferiority of the 1-day DEX regimen, whereas non-inferiority was not confirmed in patients receiving cisplatin-based chemotherapy. In a multicenter, randomized, open-designed, non-inferiority, three-arm study, Celio et al. investigated chemotherapy-naïve patients who received their first course of cisplatin-based (> 70 mg/m^2^) chemotherapy [8]. All patients received oral NEPA (netupitant 300 mg plus palonosetron 0.5 mg) and DEX 12 mg i.v. before chemotherapy and were randomized to no DEX days 2–4 (DEX1), oral DEX 4 mg × 1 days 2–3 (DEX3), or oral DEX 4 mg × 2 on days 2–4 (DEX4). The primary endpoint was CR (defined as above) in the overall phase. The study was powered (80%) not to overlook differences larger than 15%. Non-inferiority was confirmed for the DEX1 arm compared to the DEX4 arm (95% CI, −12.3 to 15%). The authors reported that a limitation was the open design and that only 33% of the patients were women. Furthermore, the overall CR in the control arm was lower (75%) than estimated in the patient sample size calculation (90%). It is important to note that none of the above studies included olanzapine as an antiemetic. It may be possible that the addition of olanzapine to a three-drug DEX-sparing regimen would be non-inferior to a conventional 4-day DEX regimen in patients treated with cisplatin. In fact the SPARED study [29] suggests that this may be possible; although results were presented as a late breaking abstract at the annual ESMO congress in 2021 [49], full publication is not available at the time of the update (September 2023).

### Olanzapine-containing regimens

Nine references evaluating olanzapine qualified for inclusion in the update. These consisted of two systematic reviews [2, 18] and seven randomized, controlled trials [11, 13, 21, 30, 38, 41, 43] of which all but one [43] used a double-blind design.

#### Olanzapine as an add-on to a three-drug regimen

Two large phase 3 studies compared the addition of olanzapine to the standard antiemetic regimen of a 5-HT_3_ receptor antagonist plus DEX plus an NK_1_ receptor antagonist. Navari et al. completed a randomized, double-blind trial comparing a 5-HT_3_ receptor antagonist plus DEX plus aprepitant/fosaprepitant plus placebo with the same 3-drug regimen plus oral olanzapine 10 mg once daily on days 1–4 after chemotherapy. The study included 380 chemotherapy-naïve patients receiving either cisplatin-based (> 70 mg/m^2^) or AC chemotherapy [30]. Patients were stratified for sex, chemotherapy (cisplatin-based versus AC), and the specific 5-HT_3_ receptor antagonist (palonosetron, granisetron, and ondansetron). The primary end point was no nausea (defined as 0 mm on a visual analogue scale during the overall assessment period from 0 to 120 h after chemotherapy). CR (defined as no emetic episodes and no need of rescue medication from 0 to 120 h after chemotherapy), no acute nausea (0–24 h), and no delayed nausea (24–120 h) were all secondary endpoints. No nausea was significantly more frequent in the olanzapine group than in the placebo group, with no nausea rates of 74% versus 45% (0–24 h, *p* = 0.002), 42% versus 25% (24–120 h, *p* = 0.002), and 37% versus 22% (0–120 h, *p* = 0.002). Also the number of patients with CR was significantly higher in the olanzapine group (86% versus 65%, 67% versus 52%, and 64% versus 41% in the acute, delayed, and overall phases, respectively. Sedation was more frequent in the olanzapine group, but both antiemetic regimens were well tolerated. Hashimoto et al. completed a similar double-blind study in chemotherapy-naïve patients receiving cisplatin-based (> 50 mg/m^2^) chemotherapy, but used olanzapine 5 mg daily for 4 days (instead of 10 mg as in the Navari study), and all patients received palonosetron (0.75 mg × 1 i.v) as the preferred 5-HT_3_ receptor antagonist in combination with DEX and aprepitant/fosaprepitant [12, 13]. The study included 705 evaluable patients, and stratification was done for sex, dose of cisplatin, and age. The primary end point was CR in the delayed phase (24–120 h after cisplatin). Olanzapine significantly improved the number of patients with CR in the delayed phase ([79%; 95% CI 75–83] versus [66%; 95% CI 61–71], *p* < 0.0001), but also in the acute and overall phases (secondary end points). Furthermore, the number of patients obtaining complete control (defined as CR and no more than mild nausea) and total control (defined as CR and no nausea) was also significantly higher in the olanzapine group. Sedation was not significantly more frequent in the olanzapine group, and the authors concluded that this was due to the lower dose of olanzapine and the administration after dinner (instead of the usual dosing in the morning). A third, randomized, double-blind study (*n* = 208) compared olanzapine 5 mg p.o daily for 4 days with placebo in chemotherapy-naïve patients with breast cancer receiving four cycles of neoadjuvant or adjuvant AC (90%) or cyclophosphamide (non-anthracycline)-based (10%) chemotherapy. All patients, in addition to olanzapine/placebo, received aprepitant, ondansetron, and DEX. The primary end point was self-reported nausea and secondary end points were control of acute and delayed nausea and vomiting [11]. Olanzapine significantly reduced the number of patients reporting nausea during all four cycles (27.7% versus 41.3%, *p* < 0.001), whereas the number of vomiting episodes was not statistically significantly reduced. Mild sedation was more frequent in the olanzapine group (54.1% versus 40.8%, *p* < 0.001).

Finally, a randomized, open, study (*n* = 120) in chemotherapy-naïve Chinese breast cancer patients receiving neoadjuvant or adjuvant AC chemotherapy compared aprepitant, ondansetron, and DEX with or without the addition of olanzapine 10 mg p.o. once daily for 5 days [43]. The authors concluded that addition of olanzapine increased the number of patients with CR (no vomiting and no use of rescue medication), the rates of no nausea (nausea on a visual analogue scale (VAS) < 5 mm), and no significant nausea (nausea VAS < 25 mm).

#### Dose and schedule of olanzapine

The most frequent adverse effect of olanzapine is sedation which could be severe in older patients [50]. The vast majority of studies (*n* ≈ 30) have investigated olanzapine in a dose and schedule of 10 mg once daily for 4 days usually administered during daytime [10, 30, 43, 51, 52]. A number of studies (*n* ≈ 15) have investigated olanzapine in a dose of 5 mg once daily [13, 48, 53, 54], and in some of these (*n* ≈ 10), olanzapine was administered at bedtime to avoid or diminish sedation [13, 48]. A few studies (*n* ≈ 10) have compared 5 mg and 10 mg of olanzapine [21, 38, 41], but none of these studies used guideline-recommended methodology or included a sufficient number of patients in order to conclude the benefits and harms between 5 mg and 10 mg [55]. A review from 2022 concluded that the evidence for administration at bedtime remains weak [56], and still no comparisons with daytime administration have been done.

### Other issues

#### Comparison of different 5-HT_3_ receptor antagonists

Two studies compared the 5-HT_3_ receptor antagonist ramosetron with palonosetron [23] and ondansetron [26], respectively. In a single-blind non-inferiority study, 279 patients were treated with cisplatin-based (72%) or AC-based (28%) chemotherapy, and all received aprepitant (days 1–3) and DEX (days 1–4) for antiemetic protection and were randomized to ramosetron 0.3 mg i.v. or palonosetron 0.25 mg i.v. on day 1. Ramosetron was non-inferior to palonosetron, with respect to the primary end point of complete response (no emesis and no rescue antiemetics in the first 5 days after chemotherapy) and all secondary end points. No differences in adverse events were observed [23]. In another single-blind study [26] with a similar design, 299 patients treated with cisplatin-based or AC-based chemotherapy all received aprepitant and DEX and were randomized to ramosetron 0.3 mg i.v. or ondansetron 16 mg i.v. on day 1. Ramosetron was non-inferior to ondansetron, but the interpretation of the results was confounded by a significant difference in the number of women allocated to ramosetron (20.8%) and ondansetron (41.9%), because it is well known that the female sex increases the risk of CINV.

Two studies compared outcomes between granisetron and palonosetron [27, 40]. In a randomized, double-blind trial, 842 patients treated with cisplatin-based (> 50 mg/m^2^) chemotherapy all received aprepitant (days 1–3) and DEX (days 1–4) and were randomized to palonosetron 0.75 mg i.v. (day 1) or granisetron 1 mg i.v. (on day 1). The study had 90% power to detect differences larger than 10%. The primary end point was CR (no emesis and no rescue antiemetics) in the first 120 h after chemotherapy. CR was not statistically significant different between the palonosetron (65.7%) and granisetron (59.1%) arms (95% CI 1.35 (0.99–1.82), *p* = 0.0539). A number of secondary end points favored palonosetron in the delayed phase (24–120 h after cisplatin), but differences were all less than 10% [40]. A randomized, double-blind study compared the effect of palonosetron 0.75 mg i.v. on day 1 against granisetron 1 mg i.v. on day 1, with both arms combined with fosaprepitant 150 mg i.v. on day 1 and DEX days 1–3 in women with breast cancer treated with AC-based chemotherapy [27]. The study included 326 patients, and the primary end point was CR (no emesis and no rescue antiemetics) in the delayed phase (24–120 h after chemotherapy). No significant differences in CR (24–120 h) were seen (CR granisetron 60.4% versus 62.3% palonosetron, *p* = 0.8) or in acute (0–24 h) or overall CR (0–120 h). An open, randomized study with a high dropout rate (18.3%) concluded that granisetron (transdermal administration) was non-inferior to ondansetron i.v., and both arms combined with aprepitant and DEX in patients receiving highly emetogenic chemotherapy [39].

A systematic review and meta-analysis published in 2021 [20] included 12 studies and concluded that palonosetron was superior to granisetron, but in a sub-analysis of the only three studies [27, 40, 57] including an NK_1_ receptor antagonist, this advantage disappeared with the exception of a minor advantage of palonosetron CR in the delayed phase (95% CI 1.30 (1.02–1.64)). However, it should be noted that olanzapine was not included in any of the above studies or in the systematic review.

#### New studies of i.v. NK_1_ receptor antagonists and comparison of different NK_1_ receptor antagonists

There are differences between the intravenous formulations of the NK_1_ receptor antagonists.

An injectable emulsion of rolapitant was approved by FDA in 2017, but due to serious hypersensitivity reactions [58], the rolapitant emulsion approval was withdrawn in January 2021 [59]. Fosaprepitant was already proven non-inferior to aprepitant and described in the 2016 guidelines [2]. Non-inferiority was recently confirmed in two large studies in Chinese patients receiving HEC, primarily cisplatin-based chemotherapy [42, 60]. Fosaprepitant induces injection site reactions (ISRs) in a small number of patients, in particular those receiving AC-based chemotherapy. Another intravenous formulation of aprepitant (HTX-019, an injectable emulsion of aprepitant free of polysorbate 80)) has a lower incidence of ISRs [59, 61, 62]. In a large phase 2 study (*i* = 584), fosnetupitant (two different doses) was compared with placebo both combined with palonosetron and DEX in patients receiving cisplatin-based (> 70 mg/m^2^) chemotherapy [37]. The high dose of fosnetupitant (235 mg) significantly improved the antiemetic effect of palonosetron and DEX as compared to placebo, and no significant differences in adverse events were observed. This confirmed results from a previous study by Hesketh et al. already reviewed in the 2016 guidelines [2]. Schwartzberg and colleagues compared intravenous NEPA (fosnetupitant and i.v. palonosetron) with oral NEPA both combined with DEX in two randomized, double-blind studies in patients receiving cisplatin-based (*n* = 404) and AC-based (*n* = 402) chemotherapy, respectively [35, 36]. The primary end point was safety and tolerability, and both studies included a multiple cycle extension (*n* = 4). It was concluded that there was no difference between i.v. and oral NEPA as concerns antiemetic efficacy or safety. It is noteworthy, that no significant differences were observed in ISRs.

Three studies compared a (fos)netupitant-based regimen against a (fos)aprepitant-based antiemetic regimen [14, 28, 45]. In a large randomized, double-blind, non-inferiority, phase 3 study (*n* = 828), oral NEPA and DEX were compared to aprepitant, granisetron, and DEX in patients receiving cisplatin-based (> 50 mg/m^2^) chemotherapy [45]. The primary end point was CR (defined as no emesis and no rescue antiemetics) during the first 120 h after start of cisplatin. Non-inferiority was demonstrated for acute CR (0–24 h), delayed CR (24–120 h), overall CR (0–120 h), and for no emesis; no nausea (< 5 mm on a 0-100 mm VAS) and no significant nausea (< 25 mm) both in the acute, delayed, and overall phases. A secondary (preplanned) analysis of the Chinese subpopulation (80.6%) confirmed these results [9]. Another randomized, double-blind, non-inferiority, phase 3 study (*n* = 785) compared fosnetupitant with fosaprepitant both combined with palonosetron and DEX in patients receiving cisplatin-based (> 70 mg/m^2^) chemotherapy [14]. Non-inferiority was proven for all efficacy end points. There were no differences in adverse effects with the exception of ISR, which was more frequently observed with fosaprepitant. Finally, a small randomized, double-blind, phase 3 study (*n* = 102) compared fosnetupitant with fosaprepitant both combined with palonosetron and DEX in patients treated with AC/EC chemotherapy [28]. The primary end point was the incidence of treatment-related adverse events (TRAEs), whereas efficacy end points were secondary. No significant differences in TRAEs were seen with the exception of TRAEs relevant for ISRs observed in 0% of the fosnetupitant patients, compared to 10% of fosaprepitant patients. It should be noted that none of the above studies compared fosnetupitant with HTX-019 aprepitant emulsion that has a lower risk of ISRs than fosaprepitant [59, 61, 62].

#### Potential new antiemetics

A few studies have investigated other drugs for the protection of nausea and vomiting in HEC [4, 16, 46]. In a randomized, double-blind, placebo-controlled, dose-ranging, phase 2 study (*n* = 318), the dopamine D_3_ receptor antagonist, amisulpride, improved the antiemetic effect of ondansetron in chemotherapy-naïve patients treated with cisplatin-based (> 70 mg/m^2^) chemotherapy [16]. A single oral dose of 10 mg days 2–4 was significantly superior to placebo as concerns the primary end point, delayed CR (no emesis and no rescue antiemetics 24–120 h after start of chemotherapy) obtained in 46% versus 20% of patients (*p* = 0.002) and the secondary end point, delayed no nausea rate (< 5 mm on a 100 mm VAS) obtained in 37% versus 19% (*p* = 0.016). No significant differences in adverse effects (including sedation) was seen. An open-label study (*n* = 100, closed prematurely due to slow recruitment) investigated the antiemetic effect of the atypical tetracyclic antidepressant, mirtazapine, with affinity for multiple receptors (serotonin, histamine, adrenergic). The study indicated that mirtazapine can improve the effect of aprepitant, palonosetron, and DEX on delayed emesis in women treated with cisplatin-based chemotherapy or EC and who experienced delayed emesis in the preceding chemotherapy cycle [4].

Thalidomide was investigated in a large randomized, double-blind trial (*n* = 638) in chemotherapy-naïve patients scheduled to receive their first course of cisplatin-based (> 50 mg/m^2^) or AC/EC chemotherapy [46]. Patients received palonosetron on day 1 and DEX on days 1–4 and were randomized to oral thalidomide 100 mg twice daily on days 1–5 or placebo. The primary end point was CR (25–120 h after start of chemotherapy). Thalidomide significantly improved the rates of CR in the delayed and overall phases (76.9% versus 61.7%, *p* < 0.001 and 66.1% versus 53.3%, *p* = 0.001, respectively). Dizziness, constipation, sedation, and dry mouth were adverse events more frequently observed with thalidomide, whereas insomnia was more frequent in the placebo-treated patients.

## Discussion

This systematic review is the result of a literature search, reported in accordance with the PRISMA guidelines for systematic reviews, and the review and discussions of the references relevant for the guideline update. One face-to-face meeting and five virtual meetings provided the background for the literature review and update of the guideline recommendations. The recommendations are summarized in Table 2.

**Table 2:** 2023 updated MASCC-ESMO recommendations high emetic risk chemotherapy

Question	Recommendation	Note	Level of evidence	Grade of recommendation
How to prevent acute nausea and vomiting following non-AC chemotherapy of high emetic risk (HEC)?	A four-drug regimen including single doses of a 5-HT_3_ receptor antagonist, dexamethasone (DEX), an NK_1_ receptor antagonist (aprepitant, fosaprepitant, netupitant*, fosnetupitant* or rolapitant), and olanzapine given before chemotherapy is recommended	*Netupitant/fosnetupitant is administered with palonosetron as part of the fixed-dose combination agent NEPA	I	A
How to prevent delayed nausea and vomiting following non-AC HEC?	In patients receiving non-AC HEC treated with a combination of a 5-HT_3_ receptor antagonist, DEX*, an NK_1_ receptor antagonist**, and olanzapine to prevent acute nausea and vomiting, DEX and olanzapine on days 2 to 4 is suggested to prevent delayed nausea and vomiting (see note about DEX dosing)	* A few studies have investigated a 1-day DEX regimen as an option in cisplatin with one study demonstrating comparable efficacy between a 1-day and multi-day DEX schedules ** If aprepitant 125 mg is used on day 1, then aprepitant 80 mg × 1 should be administered on days 2–3	II	B
How to prevent acute nausea and vomiting following anthracycline-cyclophosphamide (AC)-based chemotherapy of high emetic risk?	In women treated with AC-based chemotherapy, a four-drug regimen including single doses of a 5-HT_3_ receptor antagonist, DEX, an NK_1_ receptor antagonist (aprepitant, fosaprepitant, netupitant*, fosnetupitant* or rolapitant), and olanzapine given before chemotherapy is recommended	This recommendation is based on extensive data in women treated with adjuvant AC for breast cancer *Netupitant/fosnetupitant is administered with palonosetron as part of the fixed-dose combination agent NEPA	I	A
How to prevent delayed nausea and vomiting following anthracycline-cyclophosphamide (AC)-based chemotherapy of high emetic risk?	In women treated with a combination of a 5-HT_3_ receptor antagonist, DEX, an NK_1_ receptor antagonist*, and olanzapine to prevent acute nausea and vomiting, olanzapine on days 2 to 4 is suggested to prevent delayed nausea and vomiting	This recommendation is based on extensive data in women treated with adjuvant AC for breast cancer *If aprepitant 125 mg is used on day 1, then aprepitant 80 mg × 1 should be administered on days 2–3	II	B
Which dose and schedule of olanzapine is to be preferred in the prevention of acute and delayed nausea and vomiting following chemotherapy of high emetic risk?	The best investigated dose is 10 mg. 5 mg is superior to placebo, but it is unknown if it is as effective as 10 mg, because no robust studies have compared the 5 mg and 10 mg doses. The only schedule investigated is once daily for 4 days (see note about sedation)	If sedation is a concern, a starting daily dose of 5 mg and/or administration at bedtime is an option	II	B

There is level I evidence to limit dosing of dexamethasone to day 1 after AC chemotherapy. For patients receiving cisplatin-based (and other non-AC HEC), results are inconclusive and the 2016 recommendation of a 3–4 day DEX regimen stands. None of the studies defined in the literature search included olanzapine, except for the SPARED study [29, 49]; however, at the time of this review writing (September 2023), it is published as an abstract only and therefore not considered in this update. It is possible, that the addition of olanzapine makes it possible to limit the administration of dexamethasone to day 1 also in patients receiving cisplatin-based chemotherapy [63].

The addition of olanzapine to a three-drug regimen of a 5-HT_3_ receptor antagonist an NK_1_ receptor antagonist and DEX was optional in the 2016 MASCC/ESMO guidelines. Recently, large, well-conducted studies [13, 30] delivered clear evidence that olanzapine improves outcomes of the above three-drug regimen and olanzapine is now recommended as a fixed part of a four-drug regimen. This is in line with the ASCO recommendations [17, 18]. Sedation is an adverse event and could be a problem in older patients. Therefore, lower doses of olanzapine and administration at bedtime have been investigated. Unfortunately comparative studies (of olanzapine 10 mg and 5 mg) are few and not sufficiently powered to conclude if the 5-mg dose is as effective as the 10-mg dose [64] (Table 2). No new significant differences between the 5-HT_3_ receptor antagonists have been disclosed in this review. It is possible that palonosetron exhibits a small advantage in the protection of delayed nausea and vomiting if an NK_1_ receptor antagonist is not available or affordable [20].

Across the different NK_1_ receptor antagonists, no new difference were disclosed. This means that there are minor differences in the pharmacology (e.g., half-life and risk of drug-drug interactions), but this has not resulted in major differences in the effect or tolerability. The i.v. formulations of fosnetupitant [14, 28] and the HTX-019 emulsion of aprepitant [59, 61, 62] both seem to have a very low risk of ISRs.

No new antiemetics qualified for inclusion in the guideline update. Two agents (amisulpride and mirtazapine) were investigated and seemed to possess antiemetic efficacy in HEC patients, but none of the studies included guideline-recommended antiemetic regimens [4, 16]. A third study concluded that thalidomide improves the effect palonosetron and DEX in patients treated with HEC, but again an NK_1_ receptor antagonist (or olanzapine) was not included and concerns about adverse events have been raised [65].

Finally, although not part of this review, it is concluded that in spite of the major contribution from olanzapine in reducing nausea, this adverse event remains the major CINV problem in HEC patients.
